# Coping styles mediating the relationship between perceived chronic stress and conspiracy beliefs about COVID-19

**DOI:** 10.1007/s12144-022-03625-7

**Published:** 2022-08-16

**Authors:** Bettina Pfeffer, Andreas Goreis, Adelais Reichmann, Ines Bauda, Diana Klinger, Mercedes M. Bock, Paul L. Plener, Oswald D. Kothgassner

**Affiliations:** 1 Department of Child and Adolescent Psychiatry, Medical University of Vienna, Vienna, Austria; 2 Department of Clinical and Health Psychology, Faculty of Psychology, University of Vienna, Vienna, Austria; 3 Outpatient Unit for Research, Teaching and Practice, Faculty of Psychology, University of Vienna, Vienna, Austria; 4 Psychosocial Services Vienna, Vienna, Austria; 5 Department of Child- and Adolescent Psychiatry and Psychotherapy, Medical University of Ulm, Ulm, Germany

**Keywords:** Conspiracy beliefs, Stress coping, COVID-19, Misinformation, Chronic stress

## Abstract

As a global health crisis, COVID-19 has led to a rise in overall stress levels. Concurrently, conspiracy beliefs regarding the origin and spread of the disease have become widespread. Engaging in such beliefs can be explained as a form of coping in order to deal with elevated levels of stress. The present study investigated the indirect effects of coping strategies in the association between perceived chronic stress and COVID-related conspiracy beliefs. We report data from an online survey (*N* = 1,354 individuals: 807 female; 508 male; 8 diverse; 6 not specified; mean age 39.14 years) in German-speaking countries collected between January and March 2021. Our results indicate that people who felt more stressed were more prone to conspiracy beliefs. Coping via acceptance and self-blame was associated with decreased tendencies towards COVID-related conspiracy beliefs, while people who used denial as a strategy were more prone to these beliefs. These findings emphasize the need for stress management interventions and effective coping strategies during times of crisis in order to reduce chronic perceived stress, promote adaptive coping, and ultimately reduce conspiracy beliefs.

## Introduction

Since its outbreak in 2019, COVID-19 has quickly evolved into a pandemic and caused a global health crisis. As already seen in similar situations before (e.g., during the MERS epidemic, see Jeong et al., 2016), COVID-19 has increased overall stress levels (Marchlewska et al., 2021; Pieh et al., 2021). Major world events such as pandemics are known to cause elevated levels of psychological distress as well as feelings of uncertainty, confusion, and existential threat in the population (Georgiou et al., 2020; Swami et al., 2016; see Goreis & Voracek, 2019, for a comprehensive review). Such societal crises, defined as a rapid and impactful societal change that calls norms of conduct, established power structures, or the existence of specific groups of people into question, often cause people to look for information to understand why these negative or unexpected events occurred. These sense-making processes can increase the likelihood of turning towards conspiracy beliefs as possible explanations for a crisis (van Prooijen & Douglas, 2017).

Simultaneously to the spread of the COVID-19 pandemic, there was also a rise in conspiracy beliefs (Jutzi et al., 2020; Marchlewska et al., 2021). Conspiracy beliefs can be defined as “a subset of false beliefs in which the ultimate chaos of an event is believed to be due to a plot by multiple actors working together with a clear goal in mind, often lawfully and in secret” (Swami et al., 2014, p. 220). While previous research found perceived stress to increase people’s tendency to engage in conspiracy beliefs (see, for example, Constantinou et al., 2021; Marchlewska et al., 2021), there are also mechanisms that can buffer the effects of stress, namely coping strategies. However, it is still disputed how perceived chronic stress is associated with the tendency to endorse conspiracy beliefs in the context of the current pandemic (see Braud et al., 2021). Moreover, it is unknown if different ways to cope with stress can alter its influence on conspiracy beliefs.

Distressing experiences, especially when associated with subjective uncertainty and threat, are related to the tendency to engage in conspiracy beliefs as explanations for the distressing events. High chronic stress levels are associated with the tendency to endorse conspiracy beliefs, especially in people who use maladaptive strategies to cope with stress (Constantinou et al., 2021; Marchlewska et al., 2021). Furthermore, given the easy access to any kind of information through social media and the internet in general, the extensive and global spread of conspiracy beliefs is facilitated even more (Constantinou et al., 2021). In a similar yet converse fashion, workplace rumors (defined as unverified information that helps to make sense and manage risk in the context of danger, ambiguity, or potential threat) have been found to increase emotional exhaustion in the context of COVID-19 (Puyod & Charoensukmongkol, 2021).

Conspiracy beliefs can cause distrust in the government and political decision-making and lead to radicalization, hostility, and resistance against public health measures (Georgiou et al., 2020; van Prooijen & Acker, 2015). Engaging in conspiracy beliefs regarding the origin and spread of diseases can also cause people to turn towards potentially dangerous treatments and to reject public health measures like vaccinations, lockdowns, or quarantines, which places those individuals and the people around them at an even greater risk for contracting and spreading the disease (Constantinou et al., 2021; Georgiou et al., 2020). Current research on this topic showed that this is also the case for the COVID-19 pandemic as conspiracy beliefs reduce compliance with governmental health measures (Marinthe et al., 2020).

Experiencing stressful situations often causes individuals to respond with coping behavior, defined as “efforts, both action-oriented and intra psychic, to manage environmental and internal demands and conflicts” (Cox, 1987). Coping behaviors are generally considered adaptive when they increase the likelihood of overcoming stressful situations (see, for example, Phungsoonthorn & Charoensukmongkol, 2022; Suthatorn & Charoensukmongkol, 2022, for evidence on helpful factors when dealing with COVID-19-related stress). Different coping styles may be classified as “approach” or “avoidance” coping strategies. Approach strategies are actions that actively cope with the stressor or the stress-related emotion and are perceived as adaptive as they have higher chances of facilitating the experience of positive affect and goal attainment, whereas avoidance strategies lead to disengagement and discontinuation of goal-directed behaviors and are therefore considered maladaptive. Avoidance coping was also found to predict the belief in both context-specific and more generic conspiracy theories, whereas social support seeking (which is an approach coping strategy) decreased these beliefs (Marchlewska et al., 2021).

Engaging in conspiracy beliefs may help to regulate acute stress as simplified and seemingly rational explanations for distressing events are provided and a sense of control and predictability is reinstalled. Relying on conspiracy beliefs can itself be viewed as a form of coping as it helps to re-establish a sense of agency, regulate emotions and maintain self-esteem (Swami et al., 2016; van Prooijen et al., 2018). It can also be described as a process for the expression of negative emotions and coping with the incomprehensibility and uncontrollability of distressing situations (Constantinou et al., 2021). Therefore, endorsing conspiracy beliefs may be an interconnected response to maladaptive coping strategies, as by believing in conspiracy theories, actual confrontation with the distressing event can be avoided (Marchlewska et al., 2021).

To sum up, the COVID-19 pandemic has led to rising levels of chronic stress in many people. Furthermore, high levels of stress are associated with a higher tendency to support conspiracy beliefs, which can be explained as a way of maladaptive coping in uncertain and stressful situations. As conspiracy beliefs about the origin and spread of COVID-19 have risen since its outbreak, it is unclear how COVID-related conspiracy beliefs are related to perceived levels of chronic stress and coping mechanisms. Therefore, we hypothesized that levels of perceived chronic stress are positively correlated with COVID-related conspiracy beliefs and that coping strategies mediate this association.

- H1: We assumed a direct effect of perceived chronic stress on COVID-related conspiracy beliefs.
- H2: We assumed this effect to be mediated by coping strategies.

**Fig. 1:**
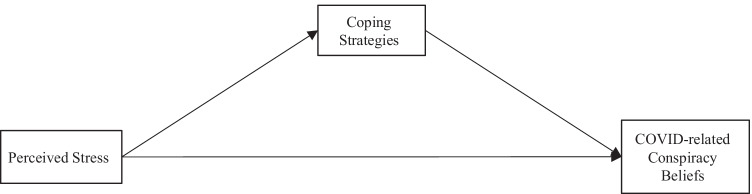
Proposed mediation model

To test these hypotheses, we proposed a mediation model with perceived chronic stress as a predictor of COVID-related conspiracy beliefs and different coping strategies as mediators (see Fig. 1 for graphical depiction).

## Methods

### Procedure and participants

The protocol for this study (no. 2171/2020) was reviewed and approved by the ethics committee of the Medical University of Vienna. Participants were recruited online via Facebook, Instagram and Reddit. In order to be included in the study, participants had to be at least 14 years of age and had to confirm their consent to the participation and the storage and utilization of their given data according to the General Data Protection Regulation (GDPR). Upon clicking the link to the study, participants were forwarded to the informed consent form, which was also available for download. The questionnaires could only be started after confirming their consent to the participation.

Data was collected from January until March 2021. A total of 1368 participants were assessed of which 39 had to be excluded due to missing values, leading to a final sample of *N* = 1329 for the present analyses (807 (60.7%) females, 508 (38.2%) males, 8 (0.6%) diverse and 6 (0.5%) not specified, age *M* = 39.14 years, *SD* = 15.166, range 14 – 93).

### Materials

#### Sociodemographic variables

The demographic data collected for descriptive use were age, gender, highest level of education, occupational status, economic status, and native language.

#### Perceived chronic stress

Subjective stress perception during the last month was assessed via the Perceived Stress Scale (Klein et al., 2016). It consists of 10 items (e.g., “In the last month, how often have you felt nervous and “stressed”?”) that are rated on a 5-point Likert scale.

#### Coping mechanisms

Individual coping strategies were assessed using the Brief Cope questionnaire (Carver, 1997). It contains 28 items (e.g., “I’ve been taking action to try to make the situation better.”) that are rated on a 5-point Likert scale. As proposed by Carver (1997), items are allocated to the subscales Active Coping, Planning, Positive Reframing, Acceptance, Humor, Religion, Using Emotional Support, Using Instrumental Support, Self-Distraction, Denial, Venting, Substance Use, Behavioral Disengagement, and Self-Blame.

#### COVID-related conspiracy beliefs

The tendency towards COVID-related conspiracy beliefs was assessed via 9 single-item questions regarding assumptions about the origin and spread of the disease (e.g., “The COVID-19 crisis is a pretext to restrict civil liberties permanently”). These assumptions were found on different internet platforms such as Twitter and also mentioned in previous research (e.g., Allington et al., 2021; Enders et al., 2020; Goreis & Kothgassner, 2020; Imhoff & Lamberty, 2020; Pummerer et al., 2022). Participants were asked to rate their agreement to each of the assumptions on a Visual Analogue Scale ranging from 0 (*not true at all*) to 100 (*completely true*). All items are listed in Appendix Table 2.

### Data analysis

Statistical Analyses were conducted using IBM SPSS Statistics 27. The mediation model was tested using the PROCESS plugin (version 3.5.3 for SPSS). A mediation model with all cope subscales as potential mediators for the effect of perceived chronic stress on COVID-related conspiracy beliefs was tested. Age and gender were included in the mediation model as covariates. Bias-corrected bootstrapping was used to test the mediating effect of the Cope subscales on the relation between perceived stress and COVID-related conspiracy beliefs. After including all subscales in one model, non-relevant mediators (with a bootstrapped CI including zero) were dropped, and relevant mediators were taken into the final mediation model. Total and direct effects were reported based on the final model. A 95% confidence interval with 5000 bootstrapping samples was used for all analyses. For result interpretation, the following general conventions were applied: an *R*^2^ of 0.02 was considered to explain a small proportion of variance, 0.13 a moderate, and 0.26 a large proportion (Cohen, 2013). Similarly, all coefficients were standardized to *z* scores before analyses, path coefficients can therefore be interpreted as correlation coefficients.

## Results

**Fig. 2:**
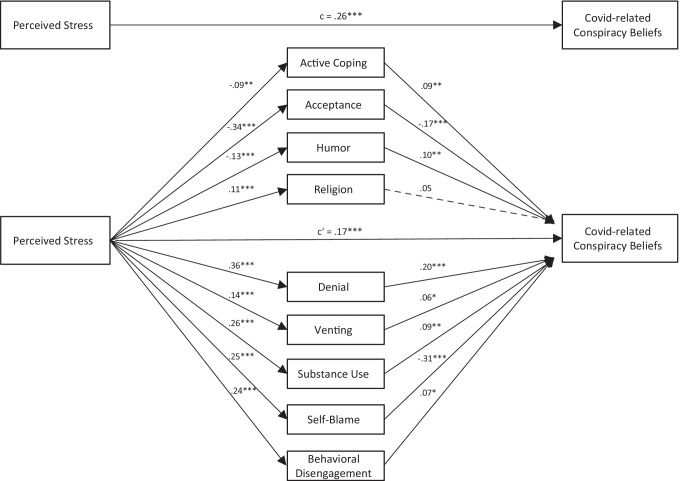
Multipath mediation model of perceived stress on COVID-related conspiracy beliefs, mediated by coping strategies. *Note*. Numbers represent unstandardized regression coefficients. Solid lines represent significant path; dashed lines represent non-significant paths. *p < .05. **p < .01. ***p < .001

All bivariate correlations of the variables included in the final model are depicted in Table 1. All path coefficients are reported as standardized coefficients. A *t*-test revealed no significant gender difference regarding the PSS sum score (*p* = 0.352). The following subscales of the Brief Cope questionnaire showed a significant mediating effect on the influence of perceived stress on COVID-related conspiracy beliefs: Active Coping, Acceptance, Humor, Religion, Denial, Venting, Substance Use, Self-Blame, and Behavioral Disengagement. Therefore, these subscales were included in the final model as mediators. The final mediation model is depicted in Fig. 2.

**Table 1:** Bivariate correlations between model variables

Variable	1	2	3	4	5	6	7	8	9	10	11	12	13
1. Perceived Stress	–												
2. Covid-related Conspiracy Beliefs	.261***	–											
3. Active Coping	-.078**	.092**	–										
4. Acceptance	-.332***	-.173***	.261***	**–**									
5. Humor	-.125***	.029	.249***	.393***	–								
6. Religion	.105***	.124***	.151***	.025	.041	–							
7. Denial	.343***	.207***	-.142***	-.385***	-.251***	.170***	–						
8. Venting	.144***	.147***	.303***	.165***	.137***	.197***	.073**	–					
9. Substance Use	.252***	.193***	-.076**	-.173***	-.020	.073**	.347***	.067*	–				
10. Behavioral Disengagement	.233***	.169***	-.040	.002	.066*	.231***	.369***	.160***	.247***	–			
11. Self-Blame	.242***	-.158***	-.085**	-.176***	-.082**	.071**	.393***	.055*	.134***	.163***	–		
12. Gender^a^	.037	.074**	.227***	.186***	.068*	-.083**	-.273***	.206***	-.152***	-.133***	-.132***	–	
13. Age	-.009	.094**	-.069*	-.175***	-.147***	.159***	.184***	-.056*	-.053	.020	-.002	-.098***	–
*M*	31,265	21,019	6,533	6,762	5,977	3,889	4,034	5,649	3,601	4,245	4,254	1,633	39,143
*SD*	7,651	27,090	2,051	2,242	2,329	2,232	2,365	2,007	2,229	1,845	2,347	0,521	15,166

1 = male, 2 = female, 3 = diverse, 4 = N/A.; *p < .05. **p < .01. ***p < .001

The total effect of perceived stress on COVID-related conspiracy beliefs was significant (c = 0.26, *p* < 0.001), the inclusion of coping strategies as mediators reduced it to a direct effect of c’ = 0.17 (*p* < 0.001). The final model explained a moderate proportion of the variance in COVID-related conspiracy beliefs (*R*^2^ = 0.23). The following mediating effects were found for the Cope subscales: perceived stress predicted Active Coping significantly (a = -0.09, *p* = 0.002), which in turn predicted COVID-related conspiracy beliefs significantly (b = 0.09, *p* = 0.001). Acceptance was significantly predicted by perceived stress (a = -0.34, *p* < 0.001) and predicted COVID-related conspiracy beliefs significantly (b = -0.17, *p* < 0.001). Perceived stress was significantly associated with Humor (a = -0.13, *p* < 0.001), which predicted COVID-related conspiracy beliefs significantly (b = 0.1, *p* = 0.001). Religion was significantly predicted by perceived stress (a = 0.11, *p* < 0.001) and showed a trend toward significance as a predictor of COVID-related conspiracy beliefs (b = 0.05, *p* = 0.056). Denial was significantly predicted by perceived stress (a = 0.36, *p* < 0.001) and in turn predicted COVID-related conspiracy beliefs significantly (b = 0.2, *p* < 0.001). Perceived stress was also a significant predictor of Venting (a = 0.14, *p* < 0.001), which predicted COVID-related conspiracy beliefs significantly (b = 0.06, *p* = 0.022). Substance Use was significantly predicted by perceived stress (a = 0.26, *p* < 0.001) and predicted COVID-related conspiracy beliefs significantly (b = 0.09, *p* = 0.001). Perceived stress predicted Behavioral Disengagement significantly (a = 0.24, *p* < 0.001), which in turn was a significant predictor of COVID-related conspiracy beliefs (a = 0.007, *p* = 0.013). Lastly, perceived stress had a significant predictive value on Self-Blame (a = 0.24, *p* < 0.001) which significantly predicted COVID-related conspiracy beliefs (a = -0.31, *p* < 0.001).

## Discussion

In this cross-sectional online study, we examined the association between perceived chronic stress, COVID-related conspiracy beliefs, and the mediating effect of coping strategies. We found a direct effect of perceived chronic stress on COVID-related conspiracy beliefs that was partially mediated by coping strategies, confirming our hypotheses. Contrary to Georgiou et al. (2020), who found no relationship between self-reported stress and COVID-related conspiracy beliefs, we found a significant effect of perceived chronic stress on COVID-related conspiracies in our sample, even after taking the mediating effects of coping strategies into account. Our findings indicate that people who reported higher stress levels in the past four weeks tend to have a stronger tendency towards conspiracy beliefs about the origin and spread of COVID-19 than those with lower levels of reported stress. There is also some debate that conspiracy beliefs may act in a bidirectional manner with perceived stress. Stress not only facilitates conspiracy beliefs but also increases feelings of helplessness and threat, which in turn increase stress levels even further (van Prooijen, 2020). However, in our study, people with high perceived chronic stress were more prone to COVID-related conspiracy beliefs, and most coping strategies – both adaptive and maladaptive – led to more COVID-related conspiracy beliefs (i.e., Active Coping, Humor, Religion, Denial, Venting, Substance Use, Behavioral Disengagement) when people perceived themselves as chronically stressed. Denial was the coping strategy that was shown to be the strongest indicator for COVID-related conspiracy beliefs. Moreover, in the present sample, self-blame as a maladaptive coping strategy and acceptance as an adaptive coping strategy were found to be negatively associated with COVID-related conspiracy beliefs. These two coping strategies seem to decrease the likelihood of believing in COVID-related conspiracies during perceived chronic stress.

Self-blame, which may be explained as “criticizing oneself for responsibility in the situation” and has been found to predict poor adjustment under stress (Carver, 1997), displayed a positive relationship with perceived chronic stress in our sample. The relation between self-blame and COVID-related conspiracy beliefs was negative, indicating that people who blame themselves in order to cope with stress have a decreased tendency to engage in COVID-related conspiracy beliefs. Concerning the compliance with governmental health measures, any coping associated with fewer conspiracy beliefs can be considered helpful or supportive for the public health situation, as engaging in conspiracy beliefs is associated with decreased measurement compliance (Constantinou et al., 2021). Still, self-blame is considered a maladaptive coping strategy for the individual and is associated with e.g. increased burnout risk, emotional exhaustion, higher posttraumatic symptomatology, and general emotional distress (Johnson & Lynch, 2013; Spataro et al., 2016). Attributing stressful events to conspiratorial explanations can also help defend people’s social identities as it allows them to blame someone else for the ongoing distressing events and protect their own in-group’s positive image. An extreme form of in-group positivity is collective narcissism, which reflects the belief in the own in-group’s greatness and is associated with defensive hostility against other groups and sensitivity to threats to the in-group’s image. People rating high on collective narcissisms are prone to conspiracy beliefs in order to protect their in-group (see Cichocka et al., 2016), which is also the case for COVID-related conspiracy beliefs (Sternisko et al., 2020), and are probably less likely to self-blame as this would harm their in-group’s image. Self-blame is a way of internal attribution, meaning that it is based on assumptions about the person. According to Weiner’s attributional theory (1985), beliefs about one’s character are global, internal, and resistant to change. Self-blame can consequently create internal negative cognitive appraisals about oneself.

The other coping strategy that has been found to decrease COVID-related conspiracy beliefs is acceptance. Acceptance has been found to predict lower distress (Carver, 1997), and it is considered a functional coping strategy, especially if the stressful situation cannot be changed and one must accommodate the circumstances (Marchlewska et al., 2021). This is the case for global crises like the COVID-19 pandemic, so that acceptance might be considered a particularly functional coping strategy during these times. Perceived chronic stress negatively predicted acceptance in our sample, indicating that people tend to use less acceptance coping under higher levels of chronic stress. Acceptance was also negatively correlated to COVID-related conspiracy beliefs, so if people used acceptance coping, they were less likely to engage in COVID-related conspiracy beliefs. Carver et al. (1989) defined denial and acceptance as polar opposites, while denial and self-blame load on the same factor (Carver, 1997). While denial predicted conspiracy beliefs positively, acceptance did so negatively, which aligns with Carver’s definition of the two coping strategies as opposites. Denial, another mediating coping strategy with a moderate positive relation with COVID-related conspiracy beliefs, is defined as “reports of refusal to believe that the stressor exists or of trying to act as though the stressor is not real” (Carver et al., 1989) and belongs to the group of maladaptive avoidance coping strategies (Marchlewska et al., 2021). Indeed, coping with denial was a predictor of distress and greater progression in somatic diseases (Carver, 1997) and it may cause additional problems if the stressor cannot be ignored (Carver et al., 1989), as is the case for a global event like COVID-19. The positive relation found in our sample indicates that people who use more denial coping are more likely to believe in COVID-related conspiracy beliefs. Similar assumptions were supported by Marchlewska et al. (2021), who found denial coping to be a predictor of conspiracy beliefs. According to our data, it seems that people, who try to push the reality of the stressful situation away are more drawn to believe in COVID-related conspiracy beliefs. Denial coping might be used as a way to avoid further confrontation with the issue as well as feeling overwhelmed with information about the event and feelings of distress. Trying to avoid cognitive dissonance arising from confrontation with contradictory information to one’s own belief systems and behaviors might also contribute to people’s tendency to use denial coping under stress. Furthermore, denying contradictory information may result in confirmation bias of the pre-existing belief systems of a person or their social bubble, which can also be reflected in echo chambers created on social media (see Goreis & Kothgassner, 2020).

The positive association between perceived chronic stress and COVID-related conspiracy beliefs implies that reduced stress might go along with decreased conspiracy beliefs, emphasizing the need for effective stress management interventions during global crises to break the potential vicious circle between chronic stress and conspiracy beliefs (see Swami et al., 2016). These interventions should focus on adaptive coping strategies associated with decreased conspiracy beliefs, as was the case for acceptance in our sample. As both denial and acceptance coping are preferably used when the distressing situation cannot be changed, interventions in such situations should focus on acceptance coping instead of denial as acceptance is not only considered a functional coping strategy (contrary to denial, which is considered maladaptive, see Marchlewska et al., 2021), but was also found to negatively predict COVID-related conspiracy beliefs in the present sample. As denial is especially used by individuals with poor problem solution strategies, these individuals might profit from enhancing self-efficacy and improving efficient problem-solving skills (Davey, 1993). To improve coping with perceived chronic stress and anxiety, promoting a safe approach to insecurity and distress as well as raising controllability awareness could be helpful as uncontrollability is known to increase stress levels (Todrank Heth & Somer, 2002). It might also be important to inform about the effects of maladaptive coping strategies such as denial and the spread of misinformation via social media to reduce conspiracy beliefs and ultimately promote compliance with public health measures. Future research might investigate the role of social media and misinformation in the spread of such conspiracy beliefs and whether enforcing controls of misinformation and fake news spread via social media might help to control the rise of conspiracy beliefs and ultimately support compliance with health measures in times of distressing global events. This might be especially important as being exposed to conspiracy beliefs has been shown to decrease the confidence in scientific consensus (van der Linden, 2015) which can be problematic when it comes to health measures based on scientific findings on disease transmission and prevention.

### Limitations

As the present research tested a mediation model that does not allow propositions regarding the direction of effects, it is possible that endorsing conspiracy beliefs also affects the use of coping strategies and levels of perceived chronic stress. The proposed model was chosen based on previous literature, but other possible cause-effect structures between the variables should also be considered. One thing to keep in mind when investigating specific, event-related conspiracy beliefs is their rapidly changing nature, so the time of data collection (in this case, January to March 2021) should be taken into account. While the theories used to assess COVID-related conspiracy beliefs were relatively widespread for the time of data collection (Allington et al., 2021; Constantinou et al., 2021; Enders et al., 2020; Goreis & Kothgassner, 2020; Imhoff & Lamberty, 2020; Pummerer et al., 2022), the specific subjects of these beliefs may change over time, so the findings from this research are limited in their generalizability to different beliefs that may have risen later.

## Conclusion

Despite these limitations, the present study provided a deeper understanding of the role of coping strategies in mediating perceived chronic stress and conspiracy beliefs regarding the origin and spread of COVID-19. We found an association between perceived chronic stress and COVID-related conspiracy beliefs as well as an influence of different coping strategies on this link. Perceived chronic stress positively predicted conspiracy beliefs, indicating that people who felt more chronically stressed were more prone to these beliefs. In addition to previous findings on the reinforcing effect of stress on conspiracy beliefs (Braud et al., 2021; Constantinou et al., 2021; Marchlewska et al., 2021), we found that different ways of coping with stress alter its effect, either increasing or decreasing the tendency to engage in conspiracy beliefs. Consequently, these findings imply the need for effective interventions to reduce stress and simultaneously support useful strategies to cope with stress as both may be associated with a lower endorsement of conspiracy beliefs.

In particular, we identified two coping strategies mediating perceived chronic stress and COVID-related conspiracy beliefs and decreasing the likelihood to believe in COVID-related conspiracy, namely self-blame and acceptance. While there was a negative mediation for self-blame and acceptance, denial positively predicted COVID-related conspiracy beliefs. This indicates that people who use denial to cope with stress are more likely to endorse COVID-related conspiracy beliefs. For clinical practice, our results imply the need for stress management interventions that promote acceptance coping to reduce COVID-related conspiracy beliefs ultimately. This is important as people who engage in these beliefs tend to neglect governmental health measures and therefore may pose a risk to public health. As conspiracy beliefs are often spread via social media, future research should also take factors like social media use, critical thinking and the awareness for misinformation and fake news into account.

## Data Availability

The datasets generated during and/or analyzed during the current study are available from the corresponding author on reasonable request.

## Appendix

Table

**Table 2:** COVID-conspiracy scale

1	COVID-19 is no worse than the common flu
2	COVID-19 crisis will be used by Bill Gates to gain more control on the world
3	COVID-19 crisis is used by secret societies to establish an authoritarian world order
4	COVID-19 is supposed to restrict civil rights and liberties permanently
5	COVID-19 is a business model for the pharmaceutical industry
6	COVID-19 crisis is used by governments to establish a dictatorship
7	COVID-19 crisis is a pretext to cover up an upcoming economic crisis
8	COVID-19 cases are artificially increased by PCR tests / PCR tests do not work and are used to artificially increase COVID-19 cases
9	COVID-19 crisis and the measures involved will be used to abrogate cash

2
